# Dry Epidermal Electrodes Can Provide Long-Term High Fidelity Electromyography for Limited Dynamic Lower Limb Movements †

**DOI:** 10.3390/s20174848

**Published:** 2020-08-27

**Authors:** Jinfeng Li, Pulin Wang, Helen J. Huang

**Affiliations:** 1 Department of Mechanical and Aerospace Engineering, University of Central Florida, Orlando, FL 32816, USA; hjhuang@ucf.edu; 2 Stretch Med, Inc., Austin, TX 78750, USA; pulin.wang@stretchmed.com; 3 Bionic Materials, Implants, and Interfaces (BiionixTM) Cluster, University of Central Florida, Orlando, FL 32816, USA

**Keywords:** surface electromyography, electrodes, dry epidermal electrodes, gold-coated, signal-to-noise ratio, signal-to-motion ratio, gait, muscle activity

## Abstract

Due to the limitations of standard wet Silver/Silver Chloride (Ag/AgCl) hydrogel electrodes and the growing demand for long-term high fidelity surface electromyography (EMG) recording, dry epidermal electrodes are of great interest. Evaluating the usability and signal fidelity of dry epidermal electrodes could help determine the extent of potential applications using EMG electrodes. We collected EMG signals over eight days from the right rectus femoris of seven subjects using single-use dry epidermal electrodes and traditional Ag/AgCl electrodes while covered and uncovered during dynamic movements (leg extension, sit-to-stand, and treadmill walking at 0.75 m/s and 1.30 m/s). We quantified signal fidelity using signal-to-noise ratio (SNR); signal-to-motion ratio (SMR); and a metric we previously developed, the Signal Quality Index, which considers that better EMG signal quality requires both good signal-to-noise ratio and good signal-to-motion ratio. Wear patterns over the eight days degraded EMG signal quality. Uncovered epidermal electrodes that remained intact and maintained good adhesion to the skin had signal-to-noise ratios, signal-to-motion ratios, and Signal Quality Index values that were above the acceptable thresholds for limited dynamic lower limb movements (leg extension and sit-to-stand). This indicated that dry epidermal electrodes could provide good signal quality across all subjects for five days for these movements. For walking, the signal-to-noise ratios of the uncovered epidermal electrodes were still above the acceptable threshold, but signal-to-motion ratios and the Signal Quality Index values were far below the acceptable thresholds. The signal quality of the epidermal electrodes that showed no visible wear was stable over five days. As expected, covering the epidermal electrodes improved signal quality, but only for limited dynamic lower limb movements. Overall, single-use dry epidermal electrodes were able to maintain high signal quality for long-term EMG recording during limited dynamic lower limb movements, but further improvement is needed to reduce motion artifacts for whole body dynamic movements such as walking.

## 1. Introduction

Myoelectrical signals measured noninvasively from superficial muscles using surface electromyography (EMG) electrodes are often used to control exoskeletons and prostheses where long-term EMG recordings would be beneficial [1,2,3]. Lower limb assistive devices such as Cyberdyne’s Hybrid Assistive Limb (HAL) exoskeleton, Ferris et al.’s pneumatically powered ankle–foot orthosis, and Au et al.’s powered ankle–foot prosthesis record EMG from at least one lower limb muscle to adjust the magnitude and timing of assistance [4,5,6,7,8,9]. How well these myoelectric controlled assistive devices function during locomotion depends on the quality of the EMG recording. Ideally, these devices would use low-surface profile EMG sensors that are not susceptible to motion artifacts and record a reliable signal from day to day, over many days [10,11]. Currently, EMG electrodes either need to be replaced after each training session or need to be cleaned regularly to maintain good signal quality, which reduces the duration that single-use electrodes can be used and affects signal stability over time [12].

There are several limitations with the most commonly used surface EMG electrodes, which typically involve wet or dry contact with the skin. The current gold standard for EMG recording is conventional Silver/Silver Chloride (Ag/AgCl) hydrogel (wet) electrodes [13,14,15], but the contact between the electrode and skin becomes inconsistent as the gel dries out over time, producing impedance variation, more motion artifacts and noise, and degraded signal quality [13]. Skin irritation caused by the gel after prolonged use also limits the Ag/AgCl electrodes to short-term use [16]. Dry electrodes can overcome some of the hurdles previously described.

Considerable progress has been made in long-term bioelectric recording. Flexible polymeric or graphene dry electrodes have been developed to monitor electrocardiographic (ECG) signals over seven days with good fidelity [17,18]. However, obtaining high-quality surface EMG is more challenging because the signal-to-noise ratio tends to be lower and EMG has a wider frequency range (5 to 500 Hz) compared to ECG (0.05 to 100 Hz) [19,20,21]. Recently, Yamagami et al. developed novel gold-based dry epidermal electrodes and validated the stability of the electrode’s surface EMG signal quality in a four-day test [22]. The dry epidermal electrodes could be removed and then reused more than 20 times. One major limitation of these electrodes, however, was that the tape became less adhesive after contact with water. Consequently, subjects had to remove the electrodes before showering or doing additional exercises.

The dry epidermal electrodes we evaluated in this study are among the few EMG electrodes that are hair-thin, skin soft, and easy to manufacture [23]. These single-use dry epidermal electrodes aim to provide reliable EMG signal recordings for at least 1 week. The electrodes use a Tegaderm transparent dressing as a substrate that makes the electrodes more skin compliant and improves the degree of adhesion, waterproofness, and breathability, enabling more continuous and multi-day use [24,25]. To our knowledge, the day-to-day EMG signal quality of single-use dry epidermal electrodes with similar features over several days has yet to be evaluated with a range of dynamic lower limb tasks that includes walking.

The objective of this study was to assess the long-term EMG recording capabilities of dry epidermal electrodes compared to traditional Ag/AgCl electrodes. We hypothesized that (1) the signal quality of the epidermal electrodes would degrade over the 8 days and (2) covering the electrodes would improve the signal quality, regardless of electrode type. This paper is an expansion of our conference paper [26] that presented preliminary data on uncovered electrodes for three individual subjects for leg extension and sit-to-stand movements. In the conference paper, we also proposed and detailed signal quality metrics that could be used to evaluate EMG signal recording quality. In this paper, we present group-level (n = 7) analyses of the EMG signal quality of the electrodes during limited dynamic lower limb movements (leg extension and sit-to-stand) and also whole body dynamic movements (treadmill walking at 0.75 m/s and 1.30 m/s). We also compared the covered and uncovered signal quality as EMG electrodes are often used underneath clothing or wrapped to the limb.

## 2. Materials and Methods

### 2.1. Electrodes

**Figure 1:**
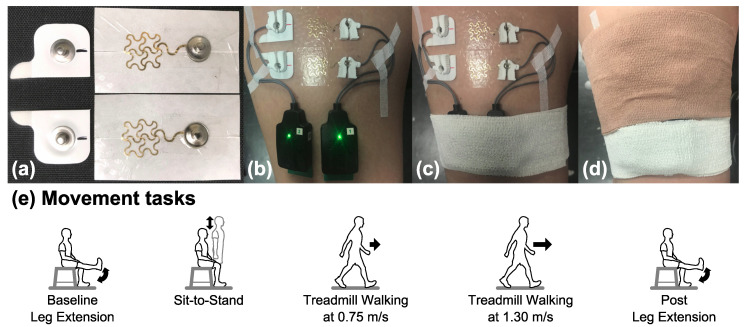
(**a**) Ag/AgCl electrodes (left) and epidermal electrodes (right). (**b**) Electrodes set-up with snap lead sensors on the right rectus femoris. (**c**) Electrodes in the uncovered setup. (**d**) Electrodes in the covered setup. (**e**) Movement tasks.

The customized dry epidermal electrode was comprised of a serpentine-shaped gold-coated polymer network (70.0 mm^2^ active area) integrated with a Tegaderm tape and had snap connectors insulated from the skin (Figure 1a). The Ag/AgCl electrode (10 mm diameter, 78.5 mm^2^ active area, Norotrode 20 Bipolar SEMG electrode) had pre-jelled electrolyte gel and also had snap connectors (Figure 1a). The snap connectors were required to use the snap–lead adapters for the Delsys Trigno IM EMG system. To help reduce crosstalk signals and obtain better signal strength [27,28], we used the shortest center-to-center interelectrode spacing possible for the epidermal electrodes, which was 30 mm. We then cut the Ag/AgCl electrodes in half so that we could increase the interelectrode spacing to 30 mm as well, which provided a more fair comparison.

### 2.2. Data Collection and Protocol

The experimental protocol was approved by the Institutional Review Board of The University of Central Florida (SBE-17-13393) and conducted in accordance with the Declaration of Helsinki. All seven (3 female, 4 male) healthy young subjects provided their written informed consent prior to participating in the experiment.

On Day 1 for a given subject, we placed the dry epidermal electrodes and arranged a setup that could be repeated from day to day for testing the two electrodes. We shaved excess body hair from right rectus femoris if necessary and gently removed the flaky skin layers with abrasive tape (3M${}^{\mathrm{TM}}$ Red Dot Trace Prep Tape). Then, we rubbed the skin with an alcohol preparation pad to remove oils and allowed the area to dry. To help prevent skin irritation, we only used soap water to clean the corresponding skin areas before applying the Ag/AgCl electrodes from Day 2 to Day 8. We used a bipolar electrode configuration and placed the Ag/AgCl electrodes and epidermal electrodes side-by-side on the right rectus femoris, following the Surface Electromyography for the Noninvasive Assessment of Muscles (SENIAM.org) guidelines (Figure 1b). We used a marker to draw landmarks on the skin of the location and orientation of the Ag/AgCl electrodes to maintain consistent placement from day to day. We placed the bodies of the two snap lead EMG sensors (Delsys, Inc., Boston, MA, USA) adjacent to each other on the distal end of right rectus femoris (Figure 1b). To minimize the movement artifact, we fixed the cables of the snap lead EMG sensors with tape (3M${}^{\mathrm{TM}}$ Cover-Roll Stretch Tape) and secured the snap lead EMG sensors with self-adhesive wrap (3M${}^{\mathrm{TM}}$ Coban Self-Adherent Wrap).

We used a Conventional Lower Body Markerset and recorded body movements at 240 Hz using a 22-camera motion capture system (OptiTrack, NaturalPoint Inc., Corvallis, OR, USA). A trigger was used to synchronize the motion capture with the EMG system that recorded the EMG signals at 2 kHz with a gain of 909 and a 20–450 Hz bandpass filter (Trigno IM system, Delsys, Inc., Boston, MA, USA).

For a given subject, the experiment started on a Monday (Day 1) and ended on the following Monday (Day 8). Day 6 and Day 7, the weekend days, were skipped. Before and after each data collection, we photographed the wear patterns of the epidermal electrodes. Subjects performed a series of movement tasks twice during each data collection, one time with the electrodes uncovered (Figure 1c) and another time with the electrodes covered using self-adherent wrap (Figure 1d). The order of the covered and uncovered conditions was balanced across subjects. We used new Ag/AgCl electrodes for each data collection while subjects kept the same epidermal electrodes on the skin continuously over eight days.

The movement tasks were (1) baseline leg extension, (2) sit-to-stand, (3) treadmill walking at 0.75 m/s, (4) treadmill walking at 1.30 m/s, and (5) post leg extension (Figure 1e). The walking tasks are whole body dynamic movements, while other tasks are limited dynamic lower limb movements. Subjects completed 20 repetitions of the limited dynamic lower limb tasks and followed a metronome to help pace subjects to perform these tasks with similar speeds. For one leg extension cycle, subjects were seated and progressed through the following movements and postures at a pace of 60 beats per minute. Each cycle had 1 high beep followed by 2 low beeps: beep 1 (high), right leg was extended where the right shank was parallel to the ground and held for 0.5 s; beep 2 (low), feet were on the ground; beep 3 (low), feet were kept on the ground before starting the next leg extension cycle at the next beep. For one sit-to-stand cycle, subjects were seated and progressed through the following movements and postures at a pace of 30 beats per minute. Each cycle had 1 high beep followed by 2 low beeps: beep 1 (high), standing upright and held for 1 s; beep 2 (low), seated again; beep 3 (low), remained seated until starting the next sit-to-stand cycle. For treadmill walking, subjects walked on a treadmill (M-gait, Motekforce Link, Amsterdam, The Netherlands) at 2 fixed speeds, 0.75 m/s and 1.30 m/s, each for two minutes. For the covered and uncovered conditions, we had the subjects do the baseline leg extension first and post leg extension last while the order of other movement tasks in between was randomized.

### 2.3. Data Analysis

We excluded data from further analysis once we found visible wear of the epidermal electrodes such as lost adhesion and contact with the skin or gold film degradation after each data collection so that comparisons were as fair as possible. We used custom MATLAB scripts (version 9.3, R2017b, MathWorks Inc., Natick, MA, USA) to process the data.

#### 2.3.1. Extraction of Movement and Gait Cycles from Motion Capture Data

We low-pass filtered the motion capture data using a 4th order Butterworth filter at 12 Hz [29] before extracting the timing of specific posture events for each task. The posture events for leg extension cycle were extension start, shank parallel to the ground, foot on the ground, and the next extension start. Extension start event was when the right heel marker’s vertical position started to increase. The shank parallel to the ground event was when the right heel marker’s vertical position reached its first peak, and the foot on the ground event was when the right heel marker’s vertical position was the lowest and closest to the ground. The events of the sit-to-stand cycle were stand up, upright, seated, to the next stand up. We defined the stand up event to be when the right thigh marker’s vertical position started to increase, the upright event to be when the right thigh marker’s vertical position reached its first peak, and the seated event to be when the right thigh marker’s vertical position was the lowest and closest to the ground. The events of the gait cycle for the treadmill walking tasks were right heel strike, left toe off, left heel strike, right toe off, and the next right heel strike. Heel strike occurred when the vertical position of the heel marker of the foot transitioning from swing to stance was near zero (close to the ground) and reached its maximum vertical acceleration [30]. Toe off occurred when the toe marker of the foot transitioning from stance to swing reached its maximum vertical acceleration between two consecutive heel strikes [31]. Using these events, we divided the EMG data into movement cycles and used the middle 16 cycles for subsequent analyses.

#### 2.3.2. EMG Analyses

In the time domain, we first high-pass filtered the EMG data using a 4th-order Butterworth filter at 40 Hz. High-pass filtering EMG data at 40 Hz is an optimal compromise between reducing motion artifacts and EMG signal amplitude loss, although it distorts the lower frequency components [32]. We then rectified the data and low-pass filtered the data with a 4th-order Butterworth filter at 40 Hz to get a linear envelope [22]. Low-pass filtering EMG data at 40 Hz preserves signal variations [22], which would be beneficial for whole body dynamic movements such as walking. To visualize the EMG time domain behavior, we averaged the linear envelopes of the first cycle for each task and electrode setup across subjects.

In the frequency domain, we computed power spectral density (PSD), signal-to-noise ratio (SNR), signal-to-motion ratio (SMR), and Signal Quality Index (SQI) using the EMG data from all cycles for each task and electrode set-up for each subject [26]. We calculated the power spectral density using the Welch’s periodogram method with 50% data overlap, and a Hamming window was applied to each segment. The detailed calculations for the signal-to-noise ratio, signal-to-motion ratio, and Signal Quality Index were reported in our previous paper [26] and will be described briefly here. The signal-to-noise ratio calculation assumed that noise present above 400 Hz [33]. We calculated the signal-to-noise ratio by dividing the total EMG power by the total noise power. A signal-to-noise ratio greater or equal to 15 dB indicated that the EMG signal was acceptable, and the signal-to-noise ratio of a simulated EMG signal would no less than 50 dB under ideal conditions [33]. Signal-to-motion ratio calculation assumed that all motion artifacts stay below 20 Hz and the uncontaminated EMG power spectrum is linear below 20 Hz [33]. The motion artifacts power was the sum of the power spectral density area below 20 Hz that exceeds a straight line between the origin and the maximum power spectral density value between 50 Hz and 150 Hz. We calculated the signal-to-motion ratio by dividing the total EMG power by the total motion artifacts power. A signal-to-motion ratio greater or equal to 12 dB indicated the EMG signal was acceptable, and the signal-to-motion ratio of a simulated EMG signal would no less than 30 dB under ideal conditions [33]. The Signal Quality Index can quantitatively and efficiently distinguish between an acceptable EMG signal (signal-to-noise ratio ≥ 15 dB, and signal-to-motion ratio ≥ 12 dB) and a contaminated EMG signal (signal-to-noise ratio < 15 dB, or signal-to-motion ratio < 12 dB) as it accounts for both the signal-to-noise ratio and signal-to-motion ratio (Equation (1)) [26].

(1) $$\mathrm{Signal}\;\mathrm{Quality}\;\mathrm{Index}\;=\;S_{1}\times S_{2}\times S_{3}$$

where

$S_{1}=(\mathrm{SNR}+\mathrm{SMR})/(15+12)$

$S_{2}=\mathrm{sgn}[(\mathrm{SNR}-15)\times (\mathrm{SMR}-12)]$

$S_{3}=10^{\left[(\mathrm{SNR}-15)+(\mathrm{SMR}-12)]/[|\mathrm{SNR}-15|+|\mathrm{SMR}-12|\right]}$

S_1_ is the scale of an acceptable signal and a contaminated signal. S_2_ is a sign index, where sgn represents sign function. S_3_ is an index between 0.1 and 10 to distinguish between an acceptable signal and a signal contaminated with low frequency artifact and high frequency noise. The changes in S_1_, S_2_, and S_3_ along with the corresponding signal-to-noise ratio and signal-to-motion ratio values are summarized in Table 1. When SNR = 15 dB and/or SMR = 12 dB, which are the acceptable threshold values, S_2_ is zero, and Signal Quality Index will be either zero or not applicable. To avoid this situation, we checked the values of SNR and SMR before calculating Signal Quality Index and increased the corresponding value of SNR or SMR by 0.001 if the SNR = 15 dB or SMR = 12 dB; this situation did not occur in this study, however.

### 2.4. Statistical Analysis

We only used the data from Day 1 to Day 5 when the epidermal electrode provided a usable signal for a subject for the nonparametric statistical analyses. To determine whether signal metrics changed over time for each electrode set-up (covered Ag/AgCl; uncovered Ag/AgCl; covered epidermal; uncovered epidermal) in each task, we performed a Friedman test on each metric (signal-to-noise ratio, signal-to-motion ratio, and Signal Quality Index) by task and with day as a factor. To determine the effects of task for each electrode setup, we also performed a Friedman test on each averaged metric by electrode setup with task as a factor. We then performed post hoc pairwise comparisons adjusted by Dunn–Bonferroni correction to identify which days or tasks were significantly different from one another. We used a Wilcoxon signed-rank test between covered Ag/AgCl and uncovered Ag/AgCl, as well as between covered epidermal and uncovered epidermal on each averaged metric by task to determine the effects of condition (covered and uncovered) on the signal metrics. We also used a Wilcoxon signed-rank test between covered Ag/AgCl and covered epidermal, as well as between uncovered Ag/AgCl and uncovered epidermal on each averaged metric by task to determine the effects of electrode type (Ag/AgCl and epidermal) on the signal metrics. We conducted the statistical analyses using SPSS software (Version 25, IBM Corp., Armonk, NY, USA) and set the significance level to 0.05.

## 3. Results

### 3.1. Electrode Wear Patterns

**Figure 2:**
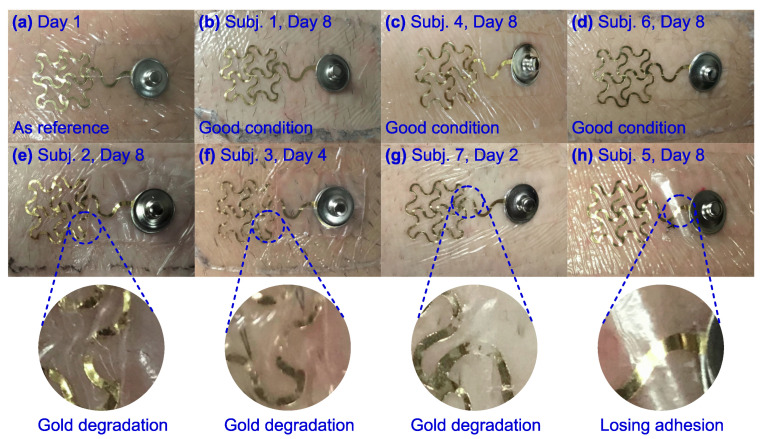
Epidermal electrodes wear patterns varied among subjects. (**a**) Day 1 as reference. (**b**) Subject 1 on Day 8. (**c**) Subject 4 on Day 8. (**d**) Subject 6 on Day 8. (**e**) Subject 2 on Day 8. (**f**) Subject 3 on Day 4. (**g**) Subject 7 on Day 2. (**h**) Subject 5 on Day 8.

The epidermal electrodes had different wear patterns among the subjects. The epidermal electrodes for Subjects 1, 4, and 6 maintained good adhesion with the skin and showed no visible gold degradation after eight days of continuous wear and looked similar to Day 1 (Figure 2a–d). The epidermal electrodes for Subjects 2, 3, and 7 showed minor gold degradation starting on Days 8, 4, and 2, respectively (Figure 2e–g). The epidermal electrodes for Subject 5 maintained good adhesion until Day 8 when the gold film started to lose adhesion (Figure 2h). Thus, Subjects 3 and 7 were excluded from future analyses as their epidermal electrodes did not have usable signals for at least 5 days.

### 3.2. EMG Signal Amplitude and Frequency Spectra Comparison

**Figure 3:**
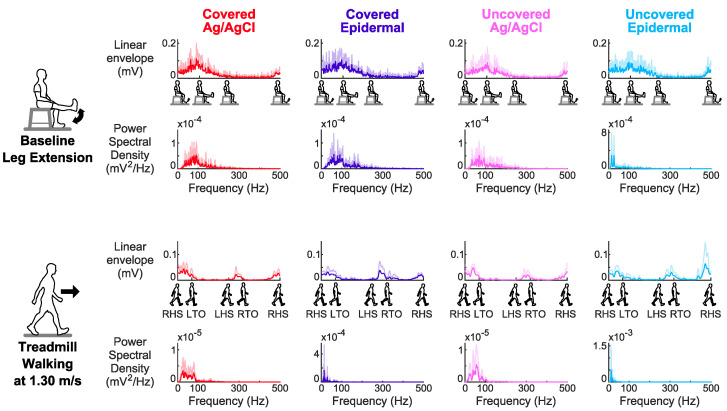
Averaged linear envelopes and power spectral densities for each electrode setup across subjects (mean + standard deviation) for the first movement cycle on Day 1 for baseline leg extension and treadmill walking at 1.30 m/s. For the linear envelopes, the movement cycles are shown on the x-axis. Baseline leg extension cycle: extension start, shank parallel to the ground, foot on the ground, and next extension start. Treadmill walking cycle: right heel strike (RHS), left toe off (LTO), left heel strike (LHS), right toe off (RTO), and next right heel strike (RHS).

For the covered and uncovered conditions, the averaged EMG signals across subjects of the Ag/AgCl and epidermal electrodes during treadmill walking at 1.30 m/s, a whole body dynamic task had different signal amplitudes and shapes in the time and frequency domains compared to baseline leg extension, a limited dynamic lower limb movement task (Figure 3).

### 3.3. Signal Stability over Days

**Figure 4:**
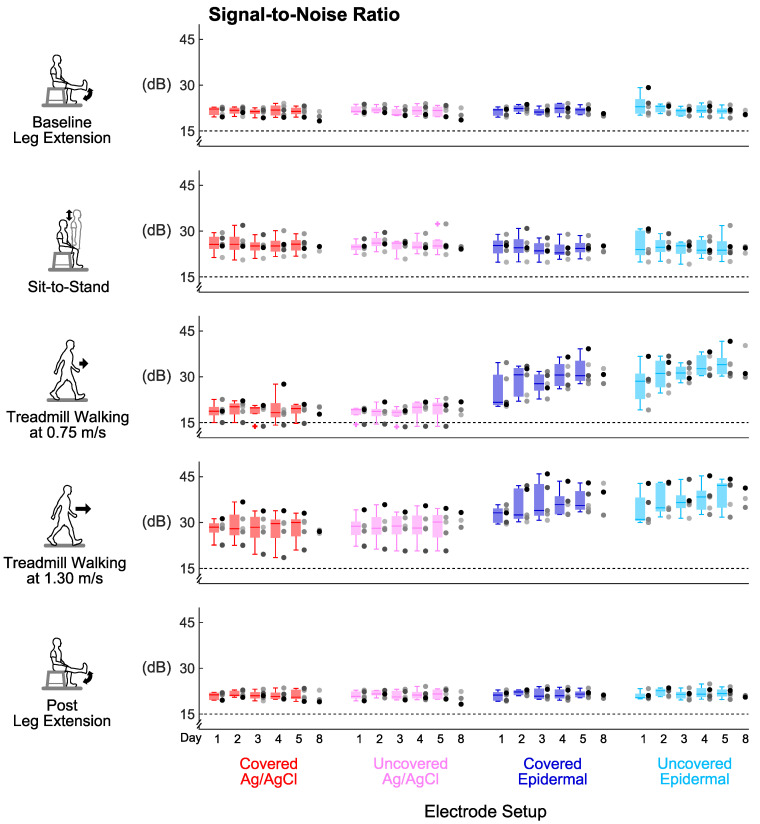
Signal stability over days: Signal-to-noise ratio of each electrode setup over 8 days in different tasks. Each dot represents individual subject (n = 5 for Days 1–5, n = 3 for Day 8), and each + represents an outlier. Dashed lines mark the 15 dB threshold for an EMG signal contaminated with noise.

**Figure 5:**
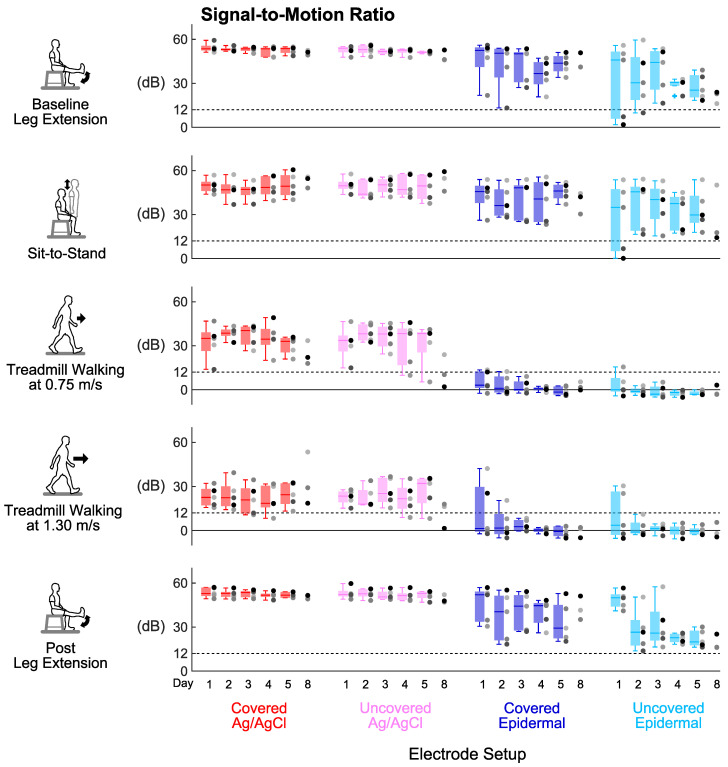
Signal stability over days: Signal-to-motion ratio of each electrode setup over 8 days in different tasks. Each dot represents individual subject (n = 5 for Days 1–5, n = 3 for Day 8), and each + represents an outlier. Dashed lines mark the 12 dB threshold for an EMG signal contaminated with motion artifacts.

**Figure 6:**
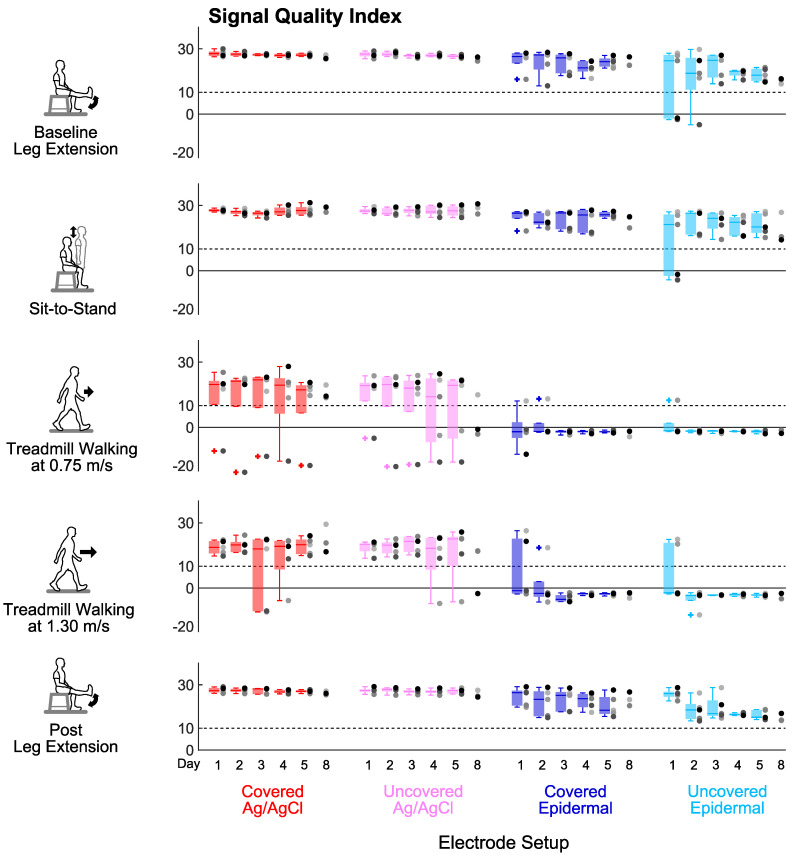
Signal stability over days: Signal Quality Index (unitless) of each electrode setup over 8 days in different tasks. Each dot represents an individual subject (n = 5 for Days 1–5, n = 3 for Day 8), and each + represents an outlier. Dashed lines mark the Signal Quality Index threshold value of 10 to help identify an acceptable signal (Signal Quality Index > 10).

Signal-to-noise ratio, signal-to-motion ratio, and Signal Quality Index were stable over the 5 days for every electrode set-up (Figure 4, Figure 5 and Figure 6). For all the tasks, day did not have a significant main effect on signal-to-noise ratio (*p’s* > 0.06) or signal-to-motion ratio (*p’s* > 0.05) of each electrode set-up. Day also did not have a significant main effect on Signal Quality Index for the covered Ag/AgCl (*p’s* > 0.09), uncovered Ag/AgCl (*p’s* > 0.1), and covered epidermal (*p’s* > 0.3) electrodes during all tasks. For the uncovered epidermal electrodes, day had a significant main effect on Signal Quality Index during treadmill walking at 1.30 m/s (*p* = 0.03), but did not have a significant main effect on Signal Quality Index during other tasks (*p’s* > 0.1). However, the Dunn–Bonferroni adjusted post hoc pairwise comparisons showed that there were no significant differences between days for the uncovered epidermal electrodes during treadmill walking at 1.30 m/s (*p’s* > 0.05).

### 3.4. Group Averaged Signal-to-Noise Ratio Results

**Figure 7:**
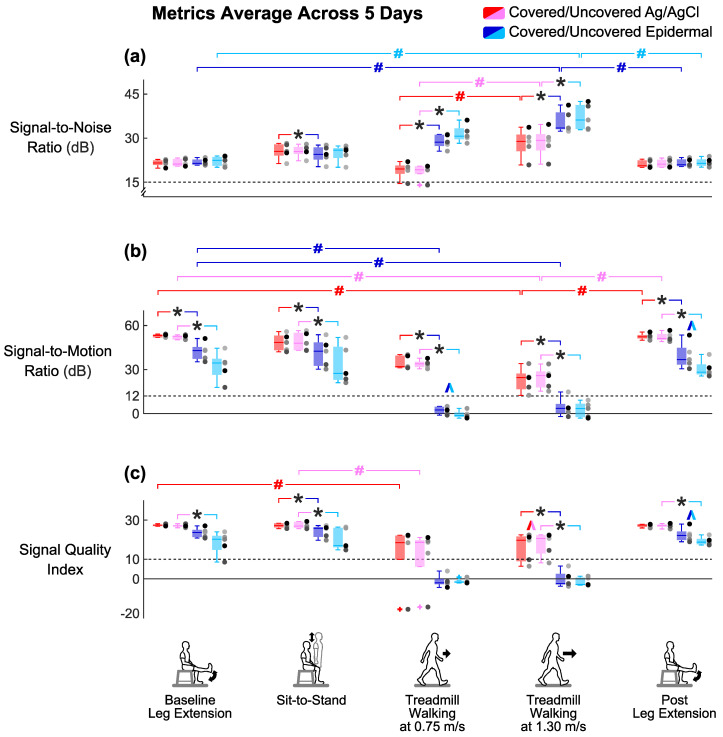
(**a**) Group averaged signal-to-noise ratio, (**b**) signal-to-motion ratio, and (**c**) Signal Quality Index across 5 days of each electrode setup for the different tasks. Each dot represents individual subject (n = 5), and each + represents an outlier. ^: significant differences between two conditions (covered and uncovered) for the same electrode type. *: significant differences between two electrode types (Ag/AgCl and epidermal) under the same condition. #: significant differences between different tasks for the same electrode setup. Colored brackets indicate the electrode setup involved in the comparison.

Covering the electrodes did not improve the signal-to-noise ratios for both electrode types compared to being uncovered (Figure 7a). Wilcoxon signed-rank tests found no significant differences in averaged signal-to-noise ratios across days either between covered Ag/AgCl and uncovered Ag/AgCl (*p’s* > 0.07) or between covered epidermal and uncovered epidermal (*p’s* > 0.07) electrodes in all tasks.

Epidermal electrodes had higher signal-to-noise ratios than Ag/AgCl electrodes during whole body dynamic movements, regardless of being covered or uncovered (Figure 7a). During both walking tasks, the averaged signal-to-noise ratios across days of the uncovered epidermal electrodes were significantly higher compared to the uncovered Ag/AgCl electrodes (*p’s* = 0.043). The median of the averaged signal-to-noise ratios across days of the walking tasks was 33.0 dB for the uncovered epidermal and 20.8 dB for the uncovered Ag/AgCl electrodes. We observed the same for the covered condition (*p’s* = 0.043), where the median of the averaged signal-to-noise ratios across days of walking tasks was 31.8 dB for the covered epidermal and 21.4 dB for the covered Ag/AgCl electrodes.

Treadmill walking at 1.30 m/s had the highest signal-to-noise ratios for every electrode setup (Figure 7a). Task had a significant main effect on averaged signal-to-noise ratios across days for each electrode setup (*p’s* < 0.02). For the epidermal electrodes, the averaged signal-to-noise ratios across days during treadmill walking at 1.30 m/s were significantly higher compared to leg extensions (*p’s* < 0.03). For the Ag/AgCl electrodes, the averaged signal-to-noise ratios across days during treadmill walking at 1.30 m/s were significantly higher compared to treadmill walking at 0.75 m/s (*p’s* = 0.027).

### 3.5. Group Averaged Signal-to-Motion Ratio Results

Covering the electrodes improved the signal-to-motion ratios for epidermal electrodes compared to the uncovered electrodes during leg extensions and sit-to-stand, the limited dynamic lower limb movements (Figure 7b). We found that the averaged signal-to-motion ratio across days of the covered epidermal electrodes was significantly higher compared to the uncovered epidermal electrodes during post leg extension (*p* = 0.043). We also found that the averaged signal-to-motion ratios across days of the covered epidermal electrodes were higher compared to the uncovered epidermal electrodes during baseline leg extension and sit-to-stand, but these were not significant (*p’s* = 0.08). Additionally, the averaged signal-to-motion ratios across days of Ag/AgCl electrodes did not significantly differ between the covered and uncovered conditions during limited dynamic lower limb movements (*p’s* > 0.2).

Epidermal electrodes had lower signal-to-motion ratios than Ag/AgCl electrodes during all tasks, regardless of covered or uncovered condition (Figure 7b). In all tasks, the averaged signal-to-motion ratios across days of the covered epidermal electrodes decreased significantly compared to the covered Ag/AgCl electrodes (*p’s* = 0.043); similarly, the averaged signal-to-motion ratios across days of the uncovered epidermal electrodes decreased significantly compared to the uncovered Ag/AgCl electrodes (*p’s* = 0.043). In addition, all the values of averaged signal-to-motion ratios across days of the epidermal electrodes were above the 12 dB acceptable threshold [33] during limited dynamic lower limb movements, with minimum and maximum values of 17.8 dB and 53.7 dB, respectively; whereas almost all the values of averaged signal-to-motion ratios across days of epidermal electrodes were below the 12 dB acceptable threshold [33] during whole body dynamic movements, with minimum and maximum values of −3.2 dB and 14.6 dB, respectively.

Treadmill walking at 1.30 m/s decreased the signal-to-motion ratios for every electrode set-up except for uncovered epidermal (Figure 7b). Task had a significant main effect on averaged signal-to-motion ratios across days for each electrode setup (*p’s* < 0.006). The averaged signal-to-motion ratios across days of the Ag/AgCl electrodes during treadmill walking at 1.30 m/s were significantly lower compared to leg extensions (*p’s* < 0.03). The averaged signal-to-motion ratios across days of the covered epidermal electrodes during whole body dynamic movements were significantly lower compared to baseline leg extension (*p’s* < 0.03). Although task had a significant main effect on averaged signal-to-motion ratio for the uncovered epidermal electrodes (*p* = 0.005), the Dunn–Bonferroni adjusted post hoc pairwise comparisons showed that there were no significant differences between tasks (*p’s* > 0.09).

### 3.6. Group Averaged Signal Quality Index Results

Covering the electrodes improved the Signal Quality Index for epidermal electrodes compared to the uncovered electrodes during leg extensions and sit-to-stand, the limited dynamic lower limb movements (Figure 7c). We found that the averaged Signal Quality Index across days of the covered epidermal electrodes was significantly higher compared to the uncovered epidermal electrodes during post leg extension (*p* = 0.043). We also found that the averaged Signal Quality Index across days of the covered epidermal electrodes was higher compared to the uncovered epidermal electrodes during baseline leg extension and sit-to-stand, but these were not significant (*p’s* = 0.08). Additionally, the averaged Signal Quality Index across days of Ag/AgCl electrodes did not significantly differ between the covered and uncovered conditions during limited dynamic lower limb movements (*p’s* > 0.2).

Epidermal electrodes had lower Signal Quality Index than Ag/AgCl electrodes under the uncovered condition during all tasks except for treadmill walking at 0.75 m/s (Figure 7c). The averaged Signal Quality Index across days of the uncovered epidermal electrodes decreased significantly compared to the uncovered Ag/AgCl electrodes during leg extensions, sit-to-stand, and treadmill walking at 1.30 m/s (*p’s* = 0.043). The averaged Signal Quality Index across days of the covered epidermal electrodes also decreased significantly compared to the covered Ag/AgCl electrodes but only during sit-to-stand and treadmill walking at 1.30 m/s (*p’s* = 0.043). In addition, almost all the values of averaged Signal Quality Index across days of the epidermal electrodes were above the Signal Quality Index threshold of 10 during limited dynamic lower limb movements, with the minimum and maximum values of 8.7 and 28.1, respectively; whereas all the values of averaged Signal Quality Index across days of epidermal electrodes were below this threshold during whole body dynamic movements, with minimum and maximum values of −4.1 and 6.7, respectively.

Treadmill walking at 1.30 m/s decreased the Signal Quality Index for the covered Ag/AgCl and covered epidermal electrodes (Figure 7c). Task had a significant main effect on averaged Signal Quality Index across days for each electrode set-up (*p’s* < 0.005). The averaged Signal Quality Index across days of the covered Ag/AgCl electrodes during treadmill walking at 0.75 m/s was significantly lower compared to baseline leg extension (*p* = 0.027). The averaged Signal Quality Index across days of the covered Ag/AgCl electrodes and covered epidermal electrodes during treadmill walking at 1.30 m/s were also lower but not significant compared to baseline leg extension (*p’s* = 0.051).

## 4. Discussion

Our primary purpose was to assess whether the long-term EMG recording capabilities of dry epidermal electrodes were as good as traditional Ag/AgCl electrodes. We found that epidermal electrodes could record high-fidelity EMG signals during limited dynamic lower limb movements (leg extensions and sit-to-stand) but not whole body dynamic tasks (walking at 0.75 m/s and 1.30 m/s). We also found that the signal quality of the epidermal electrodes was stable over the 5 days, which does not support our hypothesis that the signal quality would degrade over days. Covering the Ag/AgCl electrodes did not improve signal quality, whereas covering epidermal electrodes improved signal quality during limited dynamic lower limb movements, which partially support our hypothesis.

The appeal of epidermal electrodes is that they could provide long-term high fidelity EMG recordings, which our results suggest is possible for up to 5 days for limited dynamic lower limb movements. We found that regardless of task, each electrode set-up could provide stable, though not necessarily acceptable, signal-to-noise ratios, signal-to-motion ratios, and Signal Quality Index for at least 5 days in most subjects. During limited dynamic lower limb movements, the epidermal electrodes had averaged signal-to-noise ratios and signal-to-motion ratios across days that were above the 15 dB and 12 dB thresholds, respectively, which indicates that EMG signals had acceptable levels of noise and motion artifacts [33]. Signal-to-noise ratios and signal-to-motion ratios of an alternative type of epidermal EMG electrode that can be removed and reapplied were also shown to be consistent over four test days for upper limb tasks [22]. Similar to our study, the gold-based epidermal electrodes used in Yamagami et al. [22] during upper limb tasks also had worse signal-to-motion ratios compared to their reference electrodes, the Delsys Trigno IM sensors. However, dry electrodes based on carbon and salt can exhibit better signal-to-motion ratios than Ag/AgCl electrodes during leg extension movements [34]. These differences in electrode materials may help explain potential discrepancies in the signal-to-motion ratios of various dry electrodes. Although the signal-to-motion ratios of epidermal electrodes were sufficiently good for recording limited dynamic lower limb movements, Ag/AgCl electrodes or other dry electrodes may provide a better option for attenuating motion artifacts.

Covering EMG electrodes is a common strategy used to improve signal quality, but, in our study, this was effective for just the epidermal electrodes during just limited dynamic lower limb movements. Interestingly, covering did not help improve the signal quality of Ag/AgCl electrodes in any task. For the Ag/AgCl electrodes, we cleaned the skin each day, reducing skin impedance, which helped to reduce motion artifacts. Covering epidermal electrodes resulted in higher averaged signal-to-motion ratios across days and averaged Signal Quality Index across days during baseline leg extension, sit-to-stand, and post leg extension, indicating that the covered epidermal electrodes could record higher fidelity signals during limited dynamic lower limb movements. These results are consistent with previous studies, which showed that appropriate contact pressure between the skin and the electrode could improve signal quality in general [35,36,37]. Wrapping self-adhesive tape over the epidermal electrodes and thigh provided some pressure that was sufficient to help reduce motion artifact caused by the relative movement of the electrode with respect to the underlying skin during limited dynamic lower limb movements but was not effective enough during whole body dynamic movements where there were more motion artifacts.

The signal quality of epidermal electrodes still needs to be improved, particularly with respect to motion artifacts if it is to be used for whole body dynamic movements such as walking. While signal-to-noise ratios for the epidermal electrodes were higher than Ag/AgCl electrodes during the walking conditions, the signal-to-motion ratios for the epidermal electrodes were well below the 12 dB threshold [33], indicating the substantial presence of motion artifacts. The near zero or negative Signal Quality Index values over multiple days during treadmill walking at 1.30 m/s further highlighted that the EMG signal quality was corrupted. Other epidermal electrode designs also had lower signal-to-motion ratios during dynamic and functional upper limb movements compared to isometric contractions [22,38]. Even for the limited dynamic lower limb movements, the signal-to-motion ratios and Signal Quality Index values for the epidermal electrodes were not consistent among all subjects, unlike for the Ag/AgCl electrodes where all subjects had fairly similar signal-to-motion ratios and Signal Quality Index values. The intersubject variability of acceptable Signal Quality Index values could still pose challenges for interpreting muscle activation patterns and developing robust EMG-based devices [39,40,41]. Despite advancements in epidermal electrode designs, motion artifacts remain a significant challenge for epidermal electrodes, particularly for dynamic applications such as myoelectric control of lower limb exoskeletons and prostheses, which is an area of application where long-term EMG signals would be beneficial [42,43,44].

Walking and other dynamic lower limb movements likely increase gold degradation and motion artifacts. Dynamic movements generate more mechanical and chemical perturbations that may accelerate gold degradation in dry epidermal electrodes. The mechanical perturbations would reduce the strength of adhesion between gold and the polymer, which is already weak due to the inertness of gold [45,46]. The chemical perturbations that increase with excessive sweat and electrolytes between the skin and electrode would degrade the gold. Treating the polymer surface with plasma [45,46,47] or applying a layer of titanium coating to the polymer surface [48] before gold deposition are two options for improving gold adhesiveness to reduce the gold degradation rate. Relative movements of the electrode with the skin, which would increase with more dynamic movements, and changes in electric potential across the epidermis are two sources of motion artifacts. Using wet electrodes with conductive gels and reducing skin impedance can reduce these types of motion artifacts [40,49].

Ideally, we would have matched electrode features, such as the active area, center-to-center interelectrode spacing, and wet/dry contacts. The size of the active area of the epidermal electrode (70.0 mm^2^) and the Ag/AgCl electrode (78.5 mm^2^) was not exactly the same. Larger electrode sizes reduce impedance [40,50] and act as a low-pass filter that smooths the signal [50,51]. These combined effects can lead to higher signal-to-noise ratios [40,50] and potentially decrease signal-to-motion ratios according to EMG simulation studies [26,33]. Interelectrode spacing tends to affect EMG recordings more than electrode size [28,52]. We used the smallest possible interelectrode spacing for both electrodes to reduce crosstalk contamination [27]. Additionally, Ag/AgCl electrodes use wet gels that naturally dampen motion artifacts [36], whereas the dry electrodes are susceptible to motion artifacts and noise [53]. We did not compare our dry epidermal electrode to other commercially available dry EMG electrodes (such as Delsys’ products) because of the large mismatches in active area and interelectrode spacing. Instead, we selected a commonly used gel-based Ag/AgCl electrode because ultimately, dry epidermal EMG electrode designs should be at least equal, if not superior, to gold standard wet Ag/AgCl electrodes [13,14,15].

The limitations of this study include having a small sample size and some electrode set-up constraints that may have affected signal quality. First, this study had a small sample size of seven subjects, and, unfortunately, only five of the seven had usable data until Day 5. The epidermal electrodes were custom designed and only a small quantity of electrodes was manufactured, which was the factor that limited the number of subjects that could be tested. Another limitation was that our EMG system used snap-lead connectors, which likely introduces its own artifacts as the connector rotates relative to the snap buttons on the electrodes. Additionally, we did not measure the electrode–skin interface impedance, which could have changed and altered the EMG recording quality before visible electrode wear. This could explain instances of poorer signal quality when epidermal electrodes showed no visible wear.

## 5. Conclusions

The single-use dry epidermal electrodes that remained intact and maintained intimate contact with the skin from day to day recorded high fidelity EMG signals during limited dynamic lower limb movements. On the other hand, the signal quality of epidermal electrodes during walking, the whole body dynamic movements, did not meet thresholds that were indicative of acceptable signal quality. Covering the epidermal electrodes improved signal quality but did not help attenuate motion artifacts sufficiently, which was the main contributor to reduced signal quality. This highlights that epidermal electrodes still need improved designs to attenuate motion artifacts. Epidermal electrodes, however, produced stable signal quality for up to five days during which subjects did not need to remove and reapply the electrodes when showering or doing additional exercises. Our findings provide new insights regarding the long-term signal quality of single-use epidermal electrodes during lower limb tasks. Overall, our findings indicate that the single-use dry epidermal electrodes are promising for long-term EMG recording, but future epidermal electrodes designs need to further reduce motion artifacts and reduce intersubject variability.

## Figures and Tables

**Table 1:** Values of Signal Quality Index, S_1_, S_2_, and S_3_ corresponding to different signal quality.

Signal Quality Index	Signal Quality	S_1_	S_2_	S_3_	Signal-to-Noise Ratio SNR (dB)	Signal-to-Motion Ratio SMR (dB)
>10	Good signal, higher values were less contaminated.	>1	+1	10	>15	>12
<0	Contaminated with low frequency artifacts.	>0	−1	>0.1 and <10	>15	<12
<0	Contaminated with high frequency noise.	>0	−1	>0.1 and <10	<15	>12
>0 and <1	Contaminated with both low frequency artifacts and high frequency noise, lower values were more contaminated.	>0 and <1	+1	0.1	<15	<12
